# Chemical screening approach using single leaves identifies compounds that affect cold signaling in *Arabidopsis*

**DOI:** 10.1093/plphys/kiad280

**Published:** 2023-05-13

**Authors:** Kohei Kitawaki, Ryota Mihara, Saori Kamimura, Akito Sato, Mari Ushiyama, Yasuko Ito-Inaba, Takehito Inaba

**Affiliations:** Department of Agricultural and Environmental Sciences, Faculty of Agriculture, University of Miyazaki, Miyazaki 889-2192, Japan; Department of Agricultural and Environmental Sciences, Faculty of Agriculture, University of Miyazaki, Miyazaki 889-2192, Japan; Department of Agricultural and Environmental Sciences, Faculty of Agriculture, University of Miyazaki, Miyazaki 889-2192, Japan; Department of Agricultural and Environmental Sciences, Faculty of Agriculture, University of Miyazaki, Miyazaki 889-2192, Japan; Department of Agricultural and Environmental Sciences, Faculty of Agriculture, University of Miyazaki, Miyazaki 889-2192, Japan; Department of Agricultural and Environmental Sciences, Faculty of Agriculture, University of Miyazaki, Miyazaki 889-2192, Japan

## Abstract

The identification of chemical compounds that affect intracellular processes has greatly contributed to our understanding of plant growth and development. In most cases, these compounds have been identified in germinated seedlings. However, chemical screening using mature plants would benefit and advance our understanding of environmental responses. In this study, we developed a high-throughput screening method using single leaves of mature plants to identify small molecules that affect cold-regulated gene expression. A single excised leaf of *Arabidopsis* (*Arabidopsis thaliana*) grown in submerged cultures responded to low temperatures in terms of *COLD-REGULATED* (*COR*) gene expression. We used transgenic *Arabidopsis* harboring a *COLD-REGULATED 15A* (*COR15A*) promoter::luciferase (*COR15Apro::LUC*) construct to screen natural compounds that affect the cold induction of *COR15Apro::LUC*. This approach allowed us to identify derivatives of 1,4-naphthoquinone as specific inhibitors of *COR* gene expression. Moreover, 1,4-naphthoquinones appeared to inhibit the rapid induction of upstream *C-REPEAT BINDING FACTOR* (*CBF*) transcription factors upon exposure to low temperature, suggesting that 1,4-naphthoquinones alter upstream signaling processes. Our study offers a chemical screening scheme for identifying compounds that affect environmental responses in mature plants. This type of analysis is likely to reveal an unprecedented link between certain compounds and plant environmental responses.

## Teaser

A chemical screening approach using single leaves identifies 1,4-naphthoquinones as compounds that affect cold signaling in *Arabidopsis*.

## Introduction

Adaptation and responses to abiotic stresses, such as low temperature and drought, are indispensable for land plants to sustain life. When plants are exposed to such stresses, they remodel their transcriptome to adapt to their environment (Uemura et al. 1995; Thomashow 2001; Zhu 2001). These processes are primarily mediated by transcription factors and upstream signaling pathways. However, we are currently far behind a complete understanding of the signaling pathways involved. One good example is the response to low-temperature stress. Although it is well known that low-temperature signaling is mediated by C-repeat binding factor/dehydration-responsive element-binding (CBF/DREB) transcription factors (Shinozaki et al. 2003), low temperature sensors remain to be identified in *Arabidopsis* (*Arabidopsis thaliana*) (Guo et al. 2018). Therefore, the development of methodologies is critical for further elucidating the signaling pathways in plants.

The identification of chemical compounds that affect intracellular signaling and trafficking has greatly contributed to our understanding of developmental regulation in plants (Hicks and Raikhel 2014; Nakamichi et al. 2022). In most cases, these compounds have been identified using germinating seedlings (∼1-wk-old seedlings at best) or cell cultures (Armstrong et al. 2004; Zhang et al. 2012; Kailasam et al. 2018; Jay et al. 2019; Uehara et al. 2019; Shirakawa et al. 2021). Chemical screening is performed at this stage using a 96-well plate. However, it is challenging to adopt the same strategy for mature plants because of their body size. Since the lifetime of *Arabidopsis* is longer than 2 months, performing chemical screening using mature plant tissues would be of great advantage. The establishment of such a screening system would allow us to better understand the signaling pathways that regulate plant growth and development during the mature stage.

In the present study, we established a high-throughput screening method using single leaves of mature plants to identify small molecules that affect cold-regulated gene expression. We used transgenic *Arabidopsis* harboring the *COLD-REGULATED 15A* (*COR15A*) promoter::luciferase (*COR15Apro::LUC*) construct as a model, because it allows the monitoring of gene expression in a nondestructive manner. A single leaf excised from *COR15Apro::LUC* plants exhibited both cold-regulated and abscisic acid (ABA)-dependent induction of luciferase activity in a microwell plate. Using this system, we screened a library of ∼500 natural compounds to identify molecules that affect the cold induction of *COR15Apro::LUC*. Among the compounds identified, we found that derivatives of 1,4-naphthoquinone specifically inhibit the cold induction of *COR15Apro::LUC*. These molecules appeared to inhibit the rapid induction of upstream *CBF* transcription factors upon exposure to low temperature, suggesting that 1,4-naphthoquinones alter upstream signaling processes. Our study offers a chemical screening scheme for identifying compounds that adjust the environmental responses of developed plants. This type of analysis is likely to reveal an unprecedented link between certain compounds and plant environmental responses.

## Results

### The excised leaves of *COR15Apro:LUC* plants grown in submerged culture can be used to achieve high-throughput chemical screening

**Figure 1.**
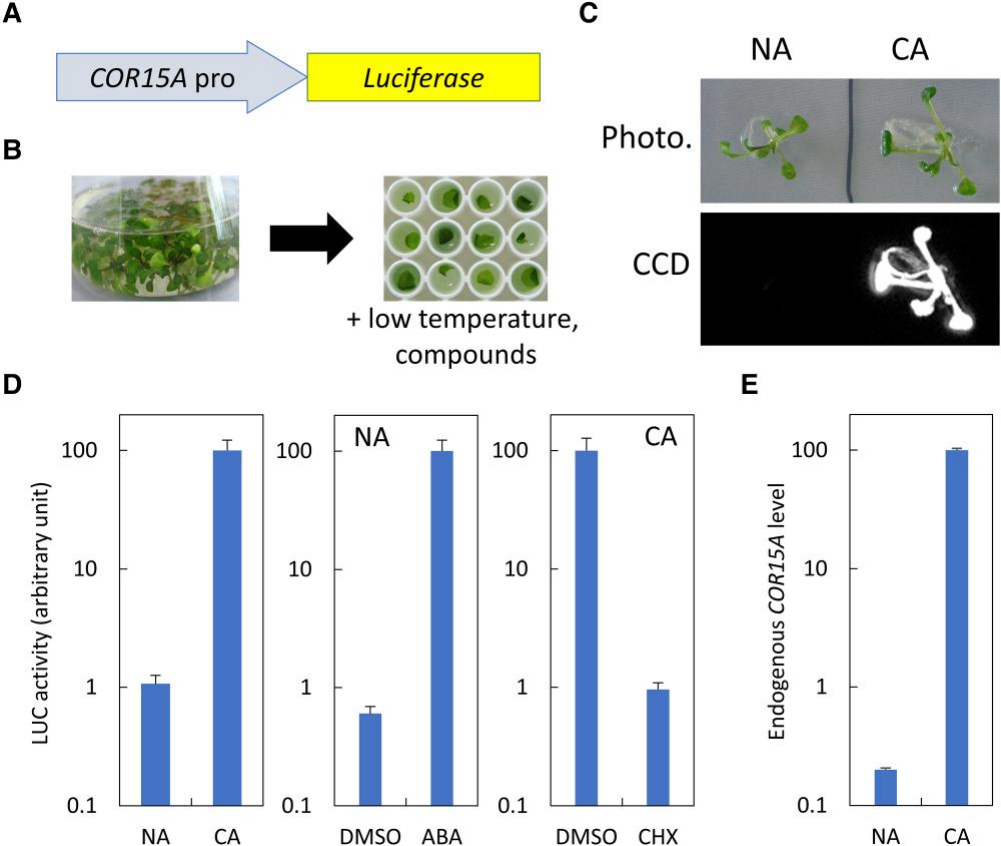
Excised leaves of *Arabidopsis* grown in submerged cultures can respond to low temperature. **A)** Schematic of the *COR15A* promoter and luciferase fusion construct (*COR15Apro::LUC*) used in this study. **B)** Plants grown in the submerged culture (left). Excised leaves were placed in a 96-well microplate (right) and treated with low temperature and/or chemical compounds. **C)** Phenotype (upper) and bioluminescence (lower) of *COR15Apro::LUC* plants grown in submerged cultures after cold acclimation (CA). A nonacclimated plant (NA) was used as the control. **D)** Excised leaves of *COR15Apro::LUC* plants grown in submerged cultures were treated with low temperature (CA in left panel) or 100 *μ*M ABA (middle panel). Plants were also treated with low temperature in the presence of DMSO or 70 *μ*M CHX (right panel). Luciferase activity was measured using a Varioskan Flash microplate reader. The bars represent Se of the mean (*n* ≥ 3). **E)** Response of endogenous *COR15A* to low temperature in leaves excised from *COR15Apro::LUC* plants grown in submerged cultures. The bars represent Se of the mean (*n* = 3).

To achieve high-throughput screening of chemical compounds in mature tissues, we first generated transgenic *Arabidopsis* carrying a luciferase reporter gene. As a model system, we selected the promoter region of *COR15A*, a well-characterized gene that is strongly induced by low-temperature treatment (Lin and Thomashow 1992; Baker et al. 1994; Nakayama et al. 2007). The 5′-upstream region of *COR15A* was fused to firefly luciferase to create the *COR15Apro::LUC* construct (Fig. 1A). We grew transgenic *Arabidopsis* carrying *COR15Apro::LUC* in submerged cultures (Fig. 1B, left) because this growth condition causes hyperhydricity of true leaves without substantial developmental effects (Ohyama et al. 2008). As visualized by a charge-coupled device (CCD) camera, *COR15Apro::LUC* plants responded to low temperatures (Fig. 1C). Furthermore, we confirmed that those plants could be cold acclimated, as they showed increased freezing tolerance (Supplemental Fig. S1).

We next examined whether excised leaves could respond similarly to low temperatures. Single leaves of *COR15Apro::LUC* plants grown in submerged cultures were excised and placed into 96-well microplates containing liquid Murashige-Skoog (MS) medium (Figs. 1B, right, and S2). The leaves were then exposed to low temperature for 48 h. As shown in Fig. 1D, the luciferase activity of cold-treated leaves was ∼100 times higher than that of nontreated leaves. We also investigated the effects of leaf size and age on cold induction of luciferase activity. Leaves that exhibited differences in size and age were selected from multiple *COR15Apro::LUC* plants grown in submerged cultures (Supplemental Fig. S2). Measurement of cold-induced luciferase activity indicated that differences in size and age did not affect low-temperature responses (Supplemental Figs. S2 and S3), confirming the stability of assay system. Although we decided to use a microplate reader for our subsequent assay, we found that a CCD camera was also applicable for the assay (Supplemental Fig. S4). We also confirmed that endogenous *COR15A* was induced under the same conditions (Fig. 1E), indicating that luciferase activity in excised *COR15Apro::LUC* leaves can be used to monitor low-temperature responses.

To further confirm that luciferase activity in excised *COR15Apro::LUC* leaves could be used for chemical screening, we treated the leaves with ABA and cycloheximide (CHX). ABA treatment induces *COR15A* expression in *Arabidopsis* plants (Baker et al. 1994), and CHX inhibits eukaryotic protein synthesis and has been shown to inhibit cold acclimation in multiple plant species (Ennis and Lubin 1964; Chen et al. 1983; Gilmour et al. 1988). Treatment of nonacclimated leaves with ABA for 4 h strongly induced luciferase activity (Fig. 1D). In contrast, when leaves were treated with CHX during low-temperature treatment, their luciferase activity after low-temperature exposure was only ∼1% that of control leaves (Fig. 1D).

These data indicate that luciferase activity in the excised leaves of *COR15Apro::LUC* plants grown in submerged cultures can be used for chemical screening. Furthermore, ABA and CHX can serve as positive controls for activation and inhibition in chemical screening, respectively.

### Screening of natural compounds that affect cold-regulated induction of *COR15Apro::LUC*

We screened 502 compounds from a natural product library (Enzo Life Sciences; Supplemental Table S1) using excised leaves from *COR15Apro::LUC* plants. Luciferase activity was measured using a Varioskan Flash microplate reader, which determined luciferase activity per leaf directly (Supplemental Fig. S2C).

**Figure 2.**
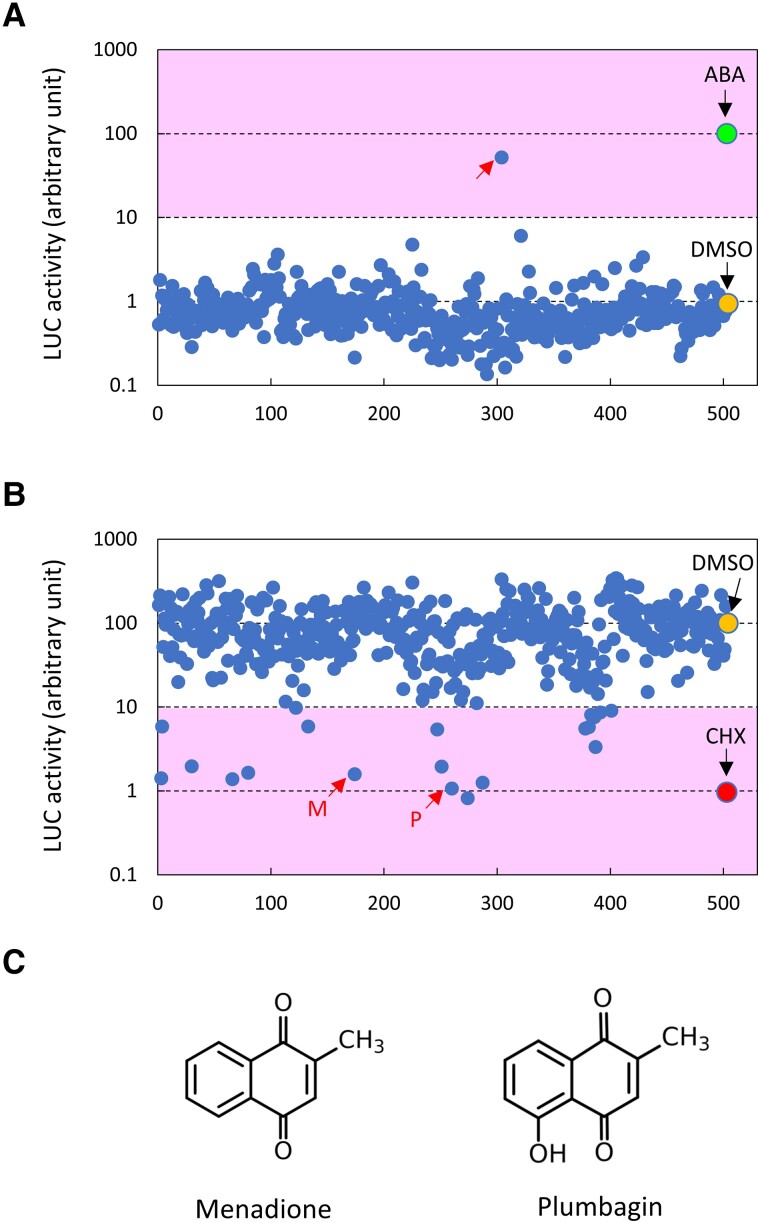
Screening of natural compounds that affect the expression of *COR15Apro::LUC*. **A)** Screening of natural compounds that induce the expression of *COR15Apro::LUC* without low-temperature treatment. Natural compounds were obtained from the Natural Product Library (ENZO), and the list is shown in Supplemental Table S1. The numbers on the *x*-axis correspond to the identification number for each compound, as shown in Supplemental Table S1; 100 *μ*M ABA and DMSO were used as positive and negative controls, respectively. Luciferase (LUC) activity in ABA-treated plants was set to 100%. A red arrow indicates ABA in the library. **B)** Screening of natural compounds that inhibit the expression of *COR15Apro::LUC* during low-temperature treatment. Screening was performed as in panel **A)**. The LUC activity in DMSO-treated plants was set to 100%; 70 *μ*M CHX and DMSO were used as positive and negative controls, respectively. Red arrows indicate menadione (M) and plumbagin (P) in the library. **C)** Chemical structures of menadione and plumbagin.

Based on the average of at least 2 independent leaves, we first searched for compounds that promoted luciferase activity without cold acclimation (Fig. 2A). ABA at a final concentration of 100 *μ*M and DMSO were used as positive and negative controls, respectively, and luciferase activity in ABA-treated leaves was set to 100%. Then, we screened for compounds inducing >10% luciferase activity at 20 *μ*g/ml concentration (50 *μ*M for a molecular weight of 400); no compound exhibiting such a level of activity was found in the library, except for ABA (Fig. 2A). This finding demonstrated that the screening system does not yield false positives and is suitable for chemical screening. We next searched for compounds that inhibited the cold induction of luciferase activity. Luciferase activity in DMSO-treated leaves exposed to low temperature was set to 100%, and CHX (70 *μ*M) was used as a positive control for inhibition. Then, we screened for compounds (20 *μ*g/ml; Supplemental Table S1) that caused at least a 90% inhibition of luciferase activity after low-temperature treatment (Fig. 2B). In the first screening round, 19 compounds met this criterion. We also included 18 additional compounds in the second screening. These 18 compounds did not meet the above criteria during the first screening; however, 1 of the tested leaves exhibited extremely low luciferase activity during the first screening. In total, we tested 37 compounds in the second screening. We measured the average of 4 independent leaves in the second screening (Supplemental Fig. S5), which allowed us to identify 4 compounds (20 *μ*g/ml concentration) that inhibited luciferase activity by >99% during cold acclimation (Supplemental Table S2).

One of these compounds was CHX (Supplemental Table S2), an inhibitor of protein synthesis, which was used as the positive control for inhibition, further confirming the validity of this screening system. Among the remaining 3 compounds, we noticed that 2 compounds, menadione and plumbagin, were 1,4-naphthoquinones and quite similar in terms of structure (Fig. 2C).

### Cold-regulated gene expression is strongly inhibited by 1,4-naphthoquinones

**Figure 3.**
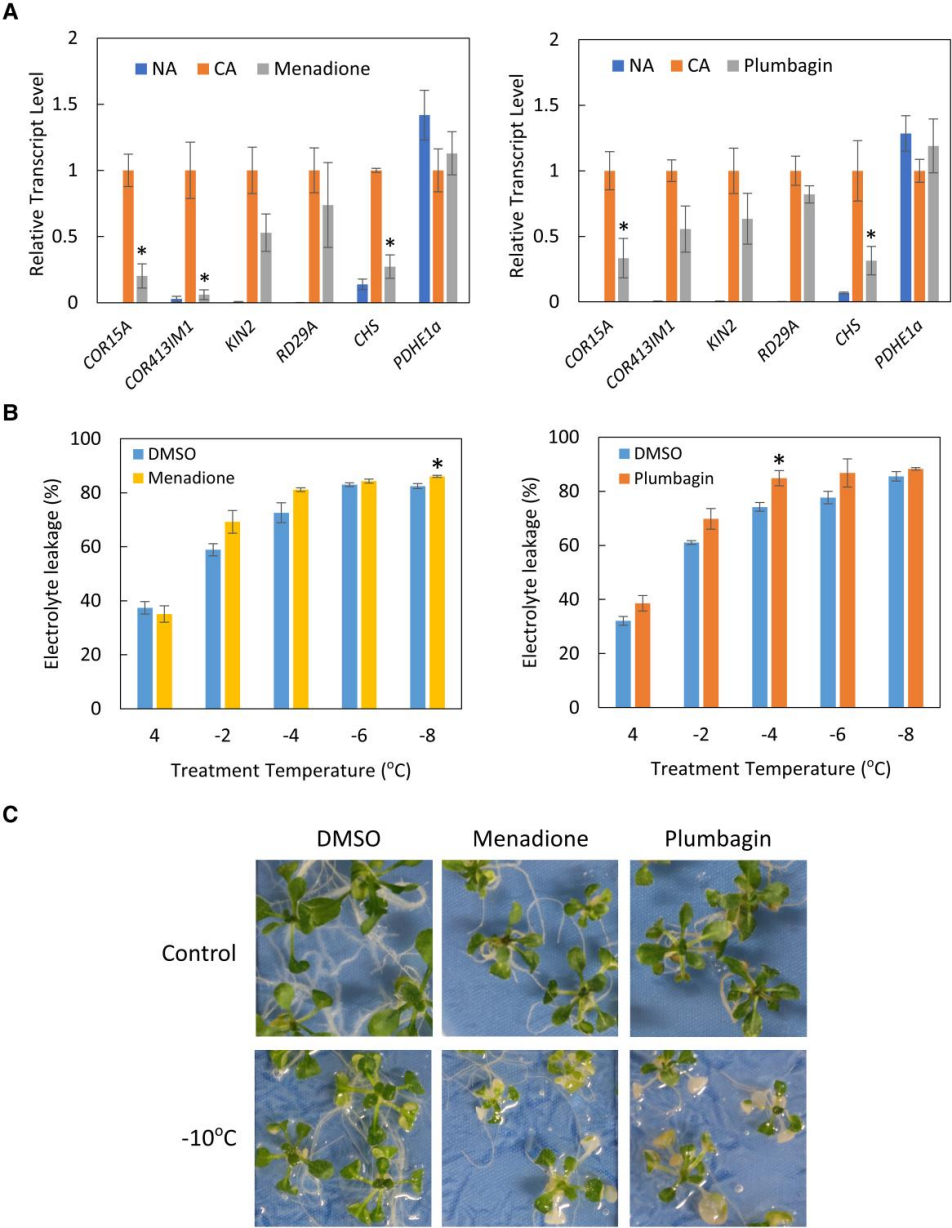
Menadione and plumbagin affect cold acclimation in *Arabidopsis*. **A)** Effects of 50 *μ*M menadione and 30 *μ*M plumbagin on cold-regulated gene expression in 2-d cold-acclimated *Arabidopsis* plants grown in submerged culture. The *PYRUVATE DEHYDROGENASE E1α SUBUNIT* (*PDHE1α*) gene was used as a control. Total RNA was extracted from true leaves, and mRNA levels were analyzed by RT-qPCR. The bars represent Se of the mean (*n* = 3). The expression levels in the cold-acclimated (CA) plants in each experimental set were set to 1. Significant differences between CA and compound-treated plants are indicated by asterisks (*P < 0.05 as determined by Student's *t*-test). **B)** Effects of 50 *μ*M menadione and 30 *μ*M plumbagin on electrolyte leakage of 2-d cold-acclimated true leaves. True leaves were excised from compound-treated plants grown in submerged cultures, placed in a test tube, and then cooled to the indicated temperatures using a programmable freezer as described in the Materials and methods. Values are the means of at least 3 independent leaves, with bars representing Se. Significant differences from DMSO-treated plants are indicated by asterisks (*P < 0.05 as determined by Student's *t*-test). **C)** Phenotype of *Arabidopsis* subjected to freezing stress. Plants were treated with DMSO, 50 *μ*M menadione or 30 *μ*M plumbagin in submerged cultures during cold acclimation and then subjected to freezing stress (−10 °C) on MS agar plates. Control plants were returned to growth chamber without freezing stress. Representative plants were selected from whole plate images shown in Supplemental Fig. S6.

To investigate whether menadione and plumbagin indeed inhibit cold-regulated gene expression, we examined the cold induction of endogenous *COR15A* in the presence of these compounds. Endogenous *COR15A* expression was strongly induced during cold acclimation. However, when the plants were treated with menadione or plumbagin during cold acclimation, the induction of endogenous *COR15A* expression was inhibited (Fig. 3A). Similar observations were made for other cold-regulated genes, such as *COLD-REGULATED 413 INNER MEMBRANE 1* (*COR413IM1*), *INDUCIBLE2* (*KIN2*), *RESPONSIVE TO DEHYDRATION 29A* (*RD29A*), and *CHALCONE SYNTHASE* (*CHS*) (Kurkela and Borg-Franck 1992; Yamaguchi-Shinozaki and Shinozaki 1993; Okawa et al. 2008), albeit to varying degrees (Fig. 3A).

We also investigated the possible effects of these compounds on the freezing tolerance of *Arabidopsis* by testing electrolyte leakage from true leaves. We examined the freezing tolerance of cold-acclimated leaves in the presence and absence of the test compounds. As shown in Fig. 3B, the overall trend was that compound-treated plants were more susceptible to freezing stress. Likewise, we also investigated the freezing tolerance of whole plants. When *Arabidopsis* plants were cold acclimated in the presence of menadione or plumbagin, both menadione- and plumbagin-treated plants were more susceptible to freezing stress (Figs. 3C and S6). Taken together, these observations are consistent with the hypothesis that menadione and plumbagin negatively regulate cold acclimation in *Arabidopsis* by affecting cold-regulated gene expression.

**Figure 4.**
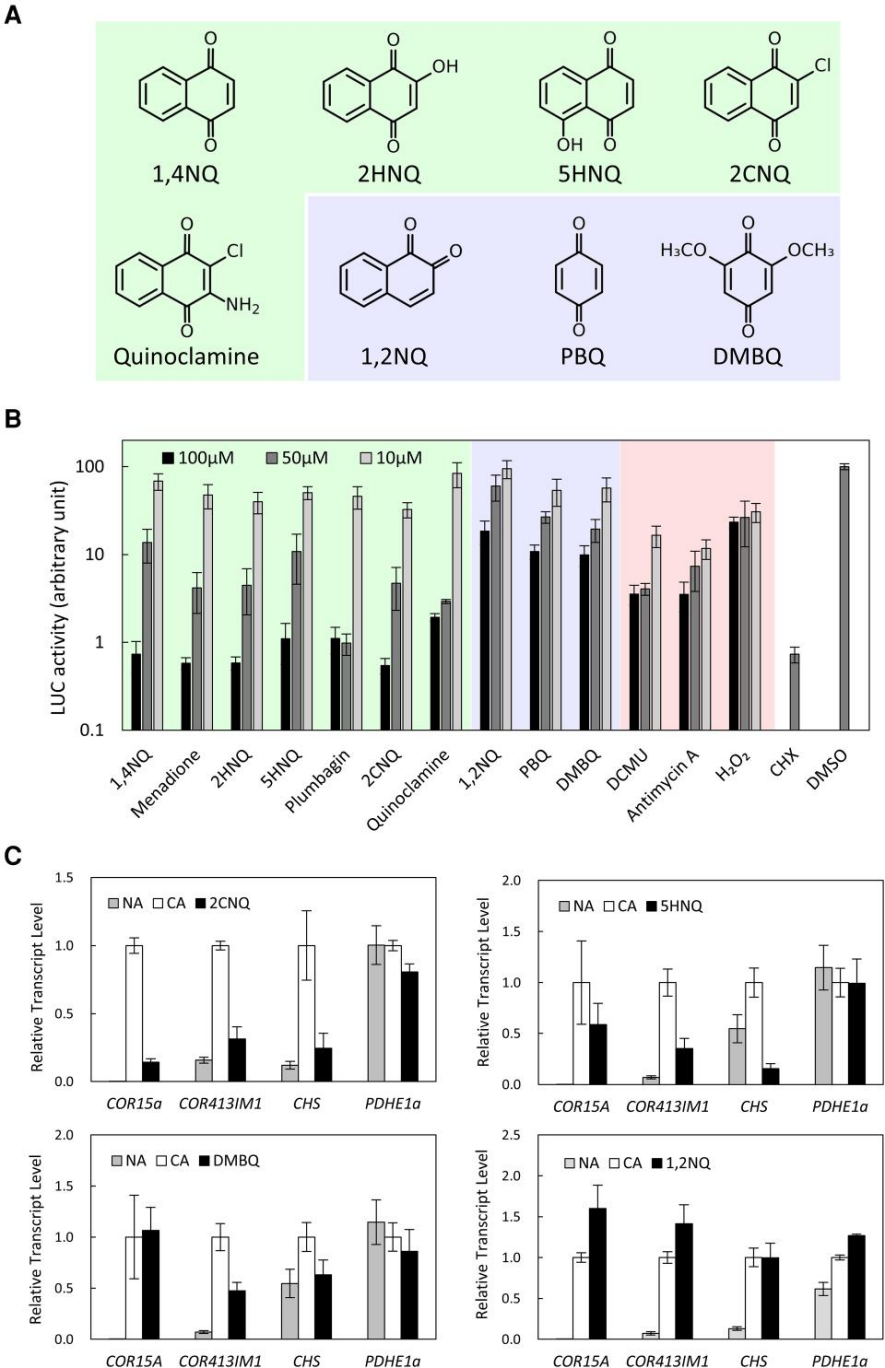
Cold-regulated gene expression is potently inhibited by 1,4-naphthoquinones. **A)** Chemical structure of the quinones used in this study. In addition to menadione and plumbagin, five 1,4-naphthoquinones were used (green). Three other quinones, 1,2-NQ, PBQ, and DMBQ were also used (purple). **B)** Effects of various quinones on *COR15Apro::LUC* expression during low-temperature treatment. Compounds that exhibited cytotoxicity similar to that of 1,4-naphthoquinones, such as DCMU, antimycin A, and H_2_O_2_, were included as controls (red). LUC activity was measured using a Varioskan Flash microplate reader. The bars represent Se of the mean (*n* = 3). **C)** The effects of 50 *μ*M quinones on the expression of cold-regulated genes in 2-d cold-acclimated wild-type *Arabidopsis* plants grown in submerged cultures. *PDHE1α* was used as control. Total RNA was extracted from true leaves, and mRNA levels were analyzed by RT-qPCR. The bars represent Se of the mean (*n* = 3). The expression levels in the cold-acclimated plants in each experimental set were set to 1. Because the 5HNQ and DMBQ treatments were performed in the same series of experiments, each graph shows the same set of CA and NA data. 1,4NQ, 1,4-naphthoquinone; 2HNQ, 2-hydroxy-1,4-naphthoquinone; 5HNQ, 5-hydroxy-1,4-naphthoquinone; 2CNQ, 2-chloro-1,4-naphthoquinone; 1,2NQ, 1,2-naphthoquinone; DMBQ, 2,6-dimethoxy-1,4-benzoquinone; PBQ, *p*-benzoquinone.

We further investigated the link between 1,4-naphthoquinones and cold-regulated gene expression in *Arabidopsis*. We investigated the effects of 10 quinone compounds on the cold induction of *COR15Apro::LUC* using excised leaves (Figs. 2C and 4A). Seven compounds were 1,4-naphthoquinones, and the other 3 compounds were quinones but not 1,4-naphthoquinones. As shown in Fig. 4B, all 7 1,4-naphthoquinones inhibited luciferase activity in a concentration-dependent manner (Fig. 4B). Using 2-chloro-1,4-naphthoquinone (2CNQ), we confirmed that difference in leaf size and age did not affect the effect of 1,4-naphthoquinones (Supplemental Figs. S2 and S3). Moreover, 1,2-naphthoquinone (1,2NQ), *p*-benzoquinone (PBQ), and 2,6-dimethoxy-1,4-benzoquinone (DMBQ) also reduced luciferase activity in a concentration-dependent manner; however, the effect was not as strong as that of 1,4-naphthoquinones (Fig. 4B).

Some 1,4-naphthoquinones exhibit cytotoxicity in vivo. Specifically, 5-hydroxy-1,4-naphthoquinone (5HNQ; known as juglone) and plumbagin inhibit electron transport in the respiratory chain and induce apoptosis in tobacco (*Nicotiana tabacum*) BY-2 cells (Babula et al. 2009). Similarly, 5HNQ and 2-hydroxy-1,4-naphthoquinone (2HNQ; also known as lawsone) induce H_2_O_2_ production and increase the activity of antioxidative enzymes (Kurtyka et al. 2016). Therefore, we examined whether compounds that exhibit similar cytotoxicity to that of 1,4-naphthoquinones, such as DCMU, antimycin A, and H_2_O_2_, could also decrease luciferase activity. DCMU is an inhibitor of photosynthetic electron transport, and antimycin A is an inhibitor of mitochondrial electron transport (Boveris and Chance 1973; Tischer and Strotmann 1977). Both DCMU and antimycin A strongly inhibited luciferase activity even at low concentrations but were less effective at higher concentrations than 1,4-naphthoquinones (Fig. 4B). The inhibitory effect of H_2_O_2_ on luciferase activity was moderate (Fig. 4B).

The effects of 1,4-naphthoquinones on cold-regulated gene expression were further evaluated using reverse transcription quantitative PCR (RT-qPCR) of endogenous genes. The expression of *COR15A*, *COR413IM1*, and *CHS* was induced by low-temperature treatment; however, this induction was inhibited by 2CNQ and 5HNQ (Fig. 4C), consistent with the observations for menadione and plumbagin (Fig. 3A). In contrast, other quinones, such as DMBQ and 1,2NQ, did not efficiently inhibit the induction of cold-regulated genes at low temperatures (Fig. 4C). Consistent with this observation, the freezing tolerance of DMBQ-treated *Arabidopsis* was comparable to that of DMSO-treated control plants (Supplemental Fig. S6). This finding also confirmed that the impaired freezing tolerance of menadione- and plumbagin-treated plants was not caused by the general cytotoxicity of quinones.

Taken together, these data suggested that 1,4-naphthoquinones specifically inhibit the cold induction of *COR* genes. Furthermore, this observation was not attributed to the cytotoxicity of 1,4-naphthoquinones.

### Rapid *CBF* induction is inhibited by 1,4-naphthoquinones

**Figure 5.**
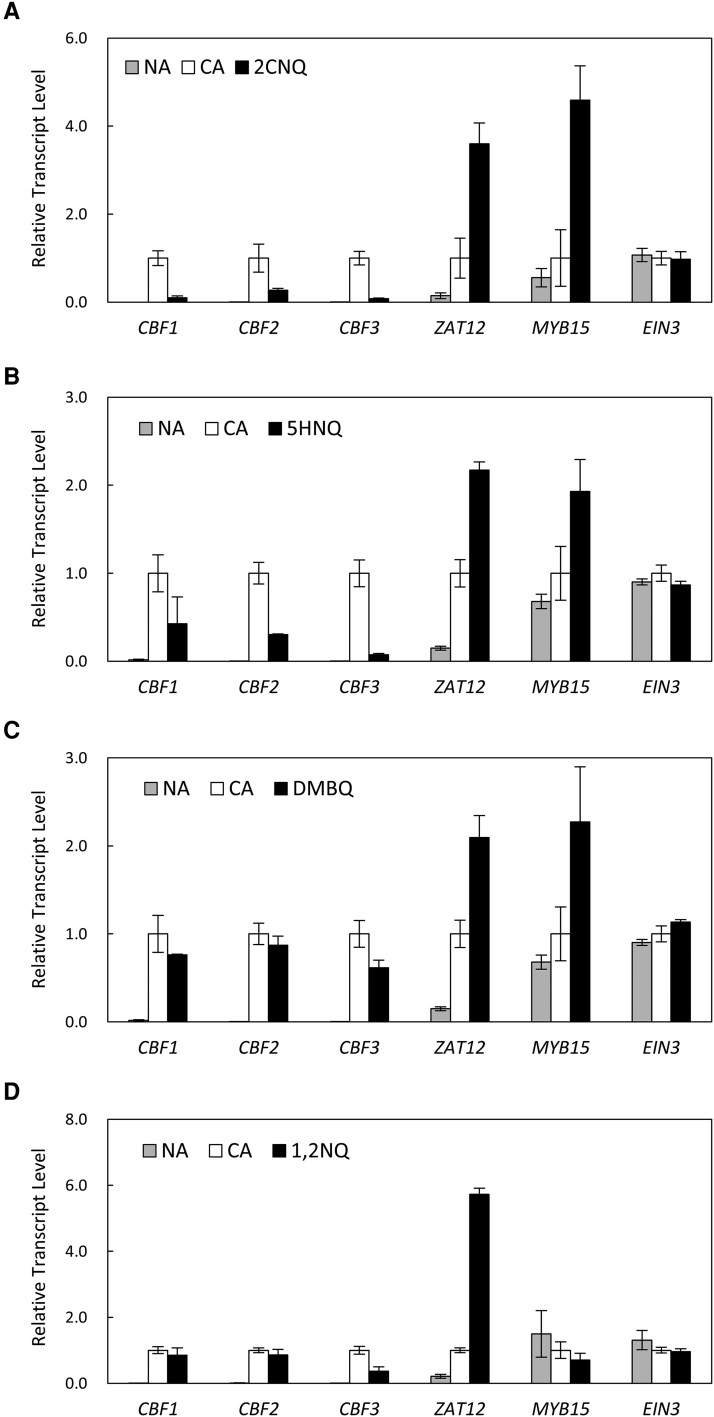
Rapid induction of *CBF* is inhibited by 1,4-naphthoquinones. *Arabidopsis* plants grown in submerged cultures for 14 d were exposed to low temperatures for 4 h in the presence or absence of 100 *μ*M 2CNQ **A)**, 5HNQ **B)**, DMBQ **C)**, and 1,2NQ **D)**. Total RNA was extracted from true leaves, and mRNA levels were analyzed by RT-qPCR. The bars represent Se of the mean (*n* = 3). The expression levels in the cold-treated plants in each experiment were set to 1. Because the 5HNQ and DMBQ treatments were performed in the same series of experiments, each graph shows the same set of CA and NA data.

Next, we investigated the possible mechanism whereby 1,4-naphthoquinones inhibited the induction of cold-regulated genes. CBF is a member of the APETALA2/Ethylene Responsive Factor (AP2/ERF) transcription factor family and is a key regulator of cold signaling in plants (Stockinger et al. 1997; Jaglo-Ottosen et al. 1998). *CBF1*, *CBF2*, and *CBF3* are induced transiently and rapidly in response to low temperatures, as well as regulate the expression of *COR* genes by binding to the C-repeat/dehydration-responsive element (Gilmour et al. 1998). Therefore, we investigated the effects of 1,4-naphthoquinones on the rapid induction of *CBF* genes. When *Arabidopsis* plants were exposed to 4 °C for 4 h, *CBF* gene expression was strongly induced (Fig. 5). This effect was diminished by the simultaneous treatment with 1,4-naphthoquinones (Fig. 5, A and B). Other quinones, such as DMBQ and 1,2NQ, were ineffective at inhibiting CBF expression (Fig. 5, C and D).

We also investigated other transcription factors that might affect the expression of *CBFs*. It has been shown that the overexpression of *ZINC FINGER OF ARABIDOPSIS THALIANA 12* (*ZAT12*), *MYB DOMAIN PROTEIN 15* (*MYB15*), or *ETHYLENE-INSENSITIVE 3* (*EIN3*) impairs the cold induction of *CBF* gene expression (Vogel et al. 2005; Agarwal et al. 2006; Shi et al. 2012). In this study, the expression of *ZAT12* and *MYB15* was strongly induced by 1,4-naphthoquinones under low temperature stress (Fig. 5). However, this was also observed for the other quinones (Fig. 5). The expression of *EIN3* did not respond to either low-temperature or quinone treatment. Hence, 1,4-naphthoquinones appear to affect the expression of *CBFs* through a mechanism that is yet to be uncovered.

Taken together, these data indicated that 1,4-naphthoquinones inhibit cold signaling by affecting the induction of *CBF* genes. The data also suggested that the potential target of 1,4-naphthoquinones is the upstream component(s) involved in the perception and signaling of low temperatures.

## Discussion

In the chemical screening of *Arabidopsis*, a major approach has been to germinate plants in microplates under sterile conditions and perform compound treatment shortly after germination (Armstrong et al. 2004; Kailasam et al. 2018; Jay et al. 2019; Uehara et al. 2019; Shirakawa et al. 2021). This approach has been used not only for reporter-based identification of chemical compounds but also for the phenotype-based identification (Jay et al. 2019). However, the life cycle of *Arabidopsis* continues thereafter. Thus, it is important to screen for compounds that affect environmental adaptation at later stages. Two major problems must be addressed. The first challenge is to obtain a compound in the cell beyond the barrier of the cuticular layer. The second issue is whether excised plant tissues exhibit the same environmental signal response as that of intact plants. Overcoming these challenges can greatly increase the value of chemical biology in plant science. Here, we developed a simple and high-throughput screening method to identify chemical compounds that affect cold acclimation, using excised leaves possessing a luciferase reporter construct. We demonstrated that this method is reliable and reproducible, as evidenced by the absence of false positives when screening for compounds upregulating *COR15Apro::LUC* expression in the absence of cold exposure (Fig. 2A). Luciferase activity was quantified directly without any image processing software (Supplemental Fig. S2C). Differences in leaf size and age did not affect either the cold induction of *COR15Apro::LUC* or the effect of the inhibitor (Supplemental Figs. S3 and S4). There are 2 important aspects of this simple and highly reproducible screening method. First, plants were cultured in liquid medium before compound treatment (Fig. 1B). Second, cold-regulated gene expression in excised leaves was comparable to that in intact plants (Fig. 1E). This method is also versatile with respect to equipment. Luciferase activity can be also quantified with a CCD camera (Supplemental Fig. S4), as luciferase activity measured by CCD camera is consistent with that measured using a microplate reader (compare Supplemental Figs. S2C and S4). Overall, our method greatly facilitates the screening of chemical compounds that regulate abiotic stress responses.

Furthermore, our study provides insights into cold signaling in *Arabidopsis*. We identified chemical compounds that inhibit cold acclimation in *Arabidopsis* via chemical screening. The identification of such compounds offers a tool to further uncover the most upstream events of low-temperature signaling. Photoreceptors such as phytochromes and phototropins serve as temperature sensors (Jung et al. 2016; Legris et al. 2016; Fujii et al. 2017). However, the temperature at which cold acclimation is strongly induced is much lower than the temperature range at which phytochromes are thought to function (Uemura et al. 1995). The freezing tolerance of *Arabidopsis phot1phot2* mutants is unaffected after cold acclimation (Imai et al. 2021), suggesting that phototropins are less likely to play key roles in temperature sensing during cold acclimation. In rice (*Oryza sativa*), CHILLING-TOLERANCE DIVERGENCE 1 (COLD1) has been proposed to serve as a temperature sensor (Ma et al. 2015); however, the role of the *Arabidopsis* homolog GTG1/GTG2 in temperature sensing remains unclear (Pandey et al. 2009).

An intriguing hypothesis is that the intracellular target of 1,4-naphthoquinones is the upstream component of low-temperature signaling, including low-temperature sensors. This hypothesis is supported by the fact that the rapid induction of CBF transcription factors was effectively inhibited by 1,4-naphthoquinones but not by other quinones (Fig. 5). In a recent study, it has been shown that a leucine-rich repeat receptor kinase, CANNOT RESPOND TO DMBQ 1 (CARD1), perceives DMBQ, a benzoquinone, to trigger defense-related gene expression and immune response to bacterial pathogens (Laohavisit et al. 2020). Another leucine-rich repeat receptor kinase, RECEPTOR-LIKE PROTEIN KINASE1 (RPK1), has been implicated in ABA perception at the plasma membrane (Osakabe et al. 2005; Konopka-Postupolska and Dobrowolska 2020; Shang et al. 2020). Our data also indicated that the rapid induction of *COR15Apro::LUC* by ABA was strongly inhibited by 2CNQ (Supplemental Fig. S7). Taken together, it appears that 1,4-naphthoquinones act on the upstream components at the plasma membrane, such as leucine-rich repeat receptor kinases that trigger low-temperature and ABA signaling. As such, chemical screening using our method and further analysis using related compounds can greatly facilitate the understanding of the signaling pathways of interest.

In summary, we established a high-throughput screening method using single leaves of mature plants. This method allows us to use intact tissue excised from whole plants for chemical screening and may be applicable to other plant species that cannot germinate within microwell plates under sterile conditions. To date, fewer compound screenings have been performed in plants than in mammals, partly due to the lack of sophisticated experimental systems, as new medical drugs have not yet been developed for plants. Our study offers a chemical screening scheme for identifying compounds that regulate plant growth and development.

## Materials and methods

### Construction of plant transformation vectors and *Arabidopsis* (*A. thaliana*) transformation

A schematic of the *COR15Apro::LUC* construct is shown in Fig. 1A. The *COR15A* upstream region (980 bp from the start codon) and luciferase gene were amplified by PCR using KOD DNA polymerase (TOYOBO). The primers used to amplify each fragment are listed in Supplemental Table S3. Multiple fragments were subcloned into *Hin*dIII-*Xba*I sites of a modified pBI121 vector using an In-Fusion HD Cloning Kit (Takara) to create a *COR15Apro::LUC* construct fused with the NOS terminator. The construct was introduced into *Arabidopsis* (Col-0) via *Agrobacterium*-mediated transformation, using the floral dip method (Clough and Bent 1998).

### Whole-plant submerged culture

For whole-plant submerged culture, ∼20 *Arabidopsis* seeds were directly sown in 50 mL of 0.5× MS medium containing 1% (*w*/*v*) sucrose (Tokumaru et al. 2017). To synchronize germination, flasks containing the medium and seeds were kept at 4 °C for 2 d after sowing. Plants were grown under continuous white light (60–80 *μ*mol m^−2^ s^−1^) for 13–14 d at 22 °C in a growth chamber (LHP-220S or LHP-350S, NK Systems). Whole cultures or excised leaves were treated with each compound. Typical leaves used in chemical screening are shown in Supplemental Fig. S2.

### Screening of compounds using transgenic *COR15Apro:LUC* plants

A natural product library (Supplemental Table S1) was purchased from ENZO life Sciences. Chemical screening was conducted using a 96-well microplate containing 200 *μ*L of 0.5× MS liquid medium with 0.5 mM luciferin. A single true leaf excised from plants grown in submerged cultures was placed in each well and treated with the compound, as described below. The final concentration of each compound was 20 *μ*g/mL during the first screening (Fig. 2). Luciferase activity was measured using a Varioskan Flash microplate reader (Thermo Fisher Scientific).

For the first screening of compounds that induce the expression of *COR15Apro::LUC* (Fig. 2A), the leaves were treated with each compound for 4 h at 22 °C. As positive and negative controls, 4 leaves in each microplate were treated with ABA at a final concentration of 100 *μ*M or DMSO, respectively.

For the first screening of compounds that inhibited the expression of *COR15Apro::LUC* at low temperatures (Fig. 2B), leaves were treated with compounds for 24 h at 4 °C. As positive and negative controls, 4 leaves from each microplate were treated with CHX at a final concentration of 70 *μ*M or DMSO, respectively. During the first screening, the average luciferase activity of at least 2 independent leaves was calculated to evaluate the effect of each compound.

The second screening (Supplemental Fig. S5) and screening of quinone compounds (Fig. 4B) were similar, with the exception that 4 independent leaves were used for the assays. Selection criteria of compounds for the second screening were as follows: 19 compounds that caused at least a 90% inhibition of luciferase activity after low-temperature treatment and 18 compounds that 1 of the tested leaves exhibited extremely low luciferase activity.

### RNA extraction and RT-qPCR analysis

For the experiments presented in Fig. 1, plants were grown in submerged cultures. The excised true leaves were then treated with low temperature and/or compounds in a microwell plate. The leaves were harvested and immediately frozen in liquid nitrogen.

For the experiments presented in Figs. 3 to 5, plants were grown in submerged cultures and then treated with low temperature and/or compounds in each flask for the indicated period. To cool the MS liquid medium immediately, the flasks were placed on ice-cold water for the first 30 min. After treatment, leaves were harvested and immediately frozen in liquid nitrogen.

In both cases, total RNA was isolated using RNAiso reagent (Takara) and cDNA was synthesized using the PrimeScript RT reagent kit (TaKaRa) with random hexamer and oligo(dT) primers. Then, RT-qPCR was performed on a Thermal Cycler Dice Real-Time System TP870 (TaKaRa) using TB Green Premix Ex Taq II (TaKaRa) or Brilliant III Ultra-Fast SYBR Green (Agilent) qPCR kits. Primers used for RT-qPCR are listed in Supplemental Table S4. The transcript level of each gene was normalized to that of *ACTIN2*.

### Electrolyte leakage assay

The electrolyte leakage assay was performed as described previously (Minami et al. 2015). Plants grown in submerged cultures were treated with the compounds at 4 °C for 2 d in each flask. To cool the MS liquid medium immediately, the flasks were placed on ice-cold water for the first 30 min. Prior to the freezing assay, excess MS medium was removed from the plants by immersion in ice-cold water. Electrolyte leakage from the test leaves was measured using a conductivity meter as EL_[Freezing temperature]_. To obtain values for 100% electrolyte leakage, the samples were boiled at 95 °C for 20 min. The percentage of electrolyte leakage from leaves was determined by the ratio of EL_[Freezing Temperature]_ to EL_[100]_ (EL_[Freezing temperature]_/EL_[100]_ × 100).

### Whole-plant freezing tolerance assay

Wild-type plants grown in submerged culture for 11 d were cold acclimated at 4 °C for 2 d under continuous light in the presence or absence of chemical compounds. For nonacclimated control (Supplemental Fig. S1), plants were grown in submerged culture for 13 d. Then, plants were transferred to 0.5× MS medium containing 1% (*w*/*v*) sucrose and 0.8% (*w*/*v*) agar. MS agar plates were incubated at −2 °C using a low-temperature incubator (LTI-700; Tokyo Rikakikai Co., Ltd., Tokyo, Japan). After 1 h of incubation, ice crystal formation was induced on plates using a spatula cooled with liquid nitrogen. The plates were incubated at −2 °C for another 1 h and were then cooled at a rate of −1 °C per 30 min to −10 °C. Once the temperature reached −10 °C, plates were incubated at the same temperature for another 1 h. The samples were then thawed overnight at 4 °C, returned to the growth chamber, and allowed to grow under normal conditions for ∼60 h.

### Statistical analysis

At least 3 independent biological replicates were sampled for each analysis. Statistical significance was determined using Student's *t*-test formula in Microsoft Excel. *P* < 0.05 was considered significantly different.

### Accession numbers

Sequence data from this article can be found in the Arabidopsis Genome Initiative with the following accession numbers: *COR15A* (At2g42540), *COR413IM1* (At1g29395), *KIN2* (At5g15970), *RD29A* (At5g52310), *CHS* (At5g13930), *CBF1* (At4g25490), *CBF2* (At4g25470), and *CBF3* (At4g25480).

## Supplementary Material

kiad280_Supplementary_Data

## Supplemental data

The following materials are available in the online version of this article.

**Supplemental Figure S1.** *Arabidopsis* grown in submerged cultures can cold acclimate and increase the freezing tolerance.

**Supplemental Figure S2.** Effects of leaf size on *COR15Apro::LUC* activity.

**Supplemental Figure S3.** Comparison of the effect of leaf size on the cold-induced luciferase activity in *COR15Apro::LUC* leaves using box-and-whisker plots.

**Supplemental Figure S4.** Visualization of LUC activity in excised *COR15Apro::LUC* leaves using cooled CCD camera.

**Supplemental Figure S5.** Second screening for the identification of natural compounds that inhibit the luciferase activity of *COR15Apro::LUC*.

**Supplemental Figure S6.** Freezing tolerance of *Arabidopsis* cold acclimated in the presence of DMSO, menadione, plumbagin, or DMBQ.

**Supplemental Figure S7.** Induction of *COR15Apro::LUC* activity by ABA is strongly inhibited by simultaneous treatment with 2CNQ.

**Supplemental Table S1.** Natural compounds used for screening.

**Supplemental Table S2.** List of potent inhibitors that affect cold induction of *COR15Apro:LUC*.

**Supplemental Table S3.** Primers used for construction of *COR15Apro::LUC*.

**Supplemental Table S4**. Gene-specific primers used in RT-qPCR

## References

[kiad280-B1] Agarwal M , Hao Y, Kapoor A, Dong CH, Fujii H, Zheng X, Zhu JK. A R2R3 type MYB transcription factor is involved in the cold regulation of CBF genes and in acquired freezing tolerance. J Biol Chem. 2006:281(49):37636–37645. 10.1074/jbc.M605895200 17015446

[kiad280-B2] Armstrong JI , Yuan S, Dale JM, Tanner VN, Theologis A. Identification of inhibitors of auxin transcriptional activation by means of chemical genetics in *Arabidopsis*. Proc Natl Acad Sci U S A. 2004:101(41):14978–14983. 10.1073/pnas.0404312101 15466695 PMC522024

[kiad280-B3] Babula P , Adam V, Kizek R, Sladky Z, Havel L. Naphthoquinone as allelochemical triggers of programmed cell death. Environ Exp Bot. 2009:65(2–3):330–337. 10.1016/j.envexpbot.2008.11.007

[kiad280-B4] Baker SS , Wilhelm KS, Thomashow MF. The 5′-region of *Arabidopsis* thaliana cor15a has cis-acting elements that confer cold-, drought- and ABA-regulated gene expression. Plant Mol Biol. 1994:24(5):701–713. 10.1007/BF00029852 8193295

[kiad280-B5] Boveris A , Chance B. The mitochondrial generation of hydrogen peroxide. General properties and effect of hyperbaric oxygen. Biochem J. 1973:134(3):707–716. 10.1042/bj1340707 4749271 PMC1177867

[kiad280-B6] Chen HH , Li PH, Brenner ML. Involvement of abscisic acid in potato cold acclimation. Plant Physiol. 1983:71(2):362–365. 10.1104/pp.71.2.362 16662831 PMC1066038

[kiad280-B7] Clough SJ , Bent AF. Floral dip: a simplified method for *Agrobacterium*-mediated transformation of *Arabidopsis thaliana*. Plant J. 1998:16(6):735–743. 10.1046/j.1365-313x.1998.00343.x 10069079

[kiad280-B8] Ennis HL , Lubin M. Cycloheximide: aspects of inhibition of protein synthesis in mammalian cells. Science 1964:146(3650):1474–1476. 10.1126/science.146.3650.1474 14208575

[kiad280-B9] Fujii Y , Tanaka H, Konno N, Ogasawara Y, Hamashima N, Tamura S, Hasegawa S, Hayasaki Y, Okajima K, Kodama Y. Phototropin perceives temperature based on the lifetime of its photoactivated state. Proc Natl Acad Sci U S A. 2017:114(34):9206–9211. 10.1073/pnas.1704462114 28784810 PMC5576800

[kiad280-B10] Gilmour SJ , Hajela RK, Thomashow MF. Cold acclimation in *Arabidopsis thaliana*. Plant Physiol. 1988:87(3):745–750. 10.1104/pp.87.3.745 16666219 PMC1054832

[kiad280-B11] Gilmour SJ , Zarka DG, Stockinger EJ, Salazar MP, Houghton JM, Thomashow MF. Low temperature regulation of the *Arabidopsis* CBF family of AP2 transcriptional activators as an early step in cold-induced COR gene expression. Plant J. 1998:16(4):433–442. 10.1046/j.1365-313x.1998.00310.x 9881163

[kiad280-B12] Guo X , Liu D, Chong K. Cold signaling in plants: insights into mechanisms and regulation. J Integr Plant Biol. 2018:60(9):745–756. 10.1111/jipb.12706 30094919

[kiad280-B13] Hicks GR , Raikhel NV. Plant chemical biology: are we meeting the promise? Front Plant Sci. 2014:5:455. 10.3389/fpls.2014.00455 25250041 PMC4157539

[kiad280-B14] Imai H , Kawamura Y, Nagatani A, Uemura M. Effects of the blue light–cryptochrome system on the early process of cold acclimation of *Arabidopsis thaliana*. Environ Exp Bot. 2021:183:104340. 10.1016/j.envexpbot.2020.104340

[kiad280-B15] Jaglo-Ottosen KR , Gilmour SJ, Zarka DG, Schabenberger O, Thomashow MF. *Arabidopsis CBF1* overexpression induces *COR* genes and enhances freezing tolerance. Science 1998:280(5360):104–106. 10.1126/science.280.5360.104 9525853

[kiad280-B16] Jay F , Vitel M, Brioudes F, Louis M, Knobloch T, Voinnet O. Chemical enhancers of posttranscriptional gene silencing in *Arabidopsis*. RNA 2019:25(9):1078–1090. 10.1261/rna.068627.118 31164480 PMC6800516

[kiad280-B17] Jung JH , Domijan M, Klose C, Biswas S, Ezer D, Gao M, Khattak AK, Box MS, Charoensawan V, Cortijo S, et al Phytochromes function as thermosensors in *Arabidopsis*. Science 2016:354(6314):886–889. 10.1126/science.aaf6005 27789797

[kiad280-B18] Kailasam S , Wang Y, Lo JC, Chang HF, Yeh KC. *S*-Nitrosoglutathione works downstream of nitric oxide to mediate iron-deficiency signaling in *Arabidopsis*. Plant J. 2018:94(1):157–168. 10.1111/tpj.13850 29396986

[kiad280-B19] Konopka-Postupolska D , Dobrowolska G. ABA Perception is modulated by membrane receptor-like kinases. J Exp Bot. 2020:71(4):1210–1214. 10.1093/jxb/erz531 32076729 PMC7031077

[kiad280-B20] Kurkela S , Borg-Franck M. Structure and expression of kin2, one of two cold- and ABA-induced genes of *Arabidopsis thaliana*. Plant Mol Biol. 1992:19(4):689–692. 10.1007/BF00026794 1627780

[kiad280-B21] Kurtyka R , Pokora W, Tukaj Z, Karcz W. Effects of juglone and lawsone on oxidative stress in maize coleoptile cells treated with IAA. AoB Plants 2016:8:plw073. 10.1093/aobpla/plw073 PMC5199135 27760740

[kiad280-B22] Laohavisit A , Wakatake T, Ishihama N, Mulvey H, Takizawa K, Suzuki T, Shirasu K. Quinone perception in plants via leucine-rich-repeat receptor-like kinases. Nature 2020:587(7832):92–97. 10.1038/s41586-020-2655-4 32879491

[kiad280-B23] Legris M , Klose C, Burgie ES, Rojas CC, Neme M, Hiltbrunner A, Wigge PA, Schafer E, Vierstra RD, Casal JJ. Phytochrome B integrates light and temperature signals in *Arabidopsis*. Science 2016:354(6314):897–900. 10.1126/science.aaf5656 27789798

[kiad280-B24] Lin C , Thomashow MF. DNA Sequence analysis of a complementary DNA for cold-regulated *Arabidopsis* gene *cor15* and characterization of the COR15 polypeptide. Plant Physiol. 1992:99(2):519–525. 10.1104/pp.99.2.519 16668917 PMC1080494

[kiad280-B25] Ma Y , Dai X, Xu Y, Luo W, Zheng X, Zeng D, Pan Y, Lin X, Liu H, Zhang D, et al COLD1 Confers chilling tolerance in rice. Cell 2015:160(6):1209–1221. 10.1016/j.cell.2015.01.046 25728666

[kiad280-B26] Minami A , Tominaga Y, Furuto A, Kondo M, Kawamura Y, Uemura M. *Arabidopsis* dynamin-related protein 1E in sphingolipid-enriched plasma membrane domains is associated with the development of freezing tolerance. Plant J. 2015:83(3):501–514. 10.1111/tpj.12907 26095877

[kiad280-B27] Nakamichi N , Yamaguchi J, Sato A, Fujimoto KJ, Ota E. Chemical biology to dissect molecular mechanisms underlying plant circadian clocks. New Phytol. 2022:235(4):1336–1343. 10.1111/nph.18298 35661165

[kiad280-B28] Nakayama K , Okawa K, Kakizaki T, Honma T, Itoh H, Inaba T. *Arabidopsis* Cor15am is a chloroplast stromal protein that has cryoprotective activity and forms oligomers. Plant Physiol. 2007:144(1):513–523. 10.1104/pp.106.094581 17384167 PMC1913801

[kiad280-B29] Ohyama K , Ogawa M, Matsubayashi Y. Identification of a biologically active, small, secreted peptide in *Arabidopsis* by in silico gene screening, followed by LC-MS-based structure analysis. Plant J. 2008:55(1):152–160. 10.1111/j.1365-313X.2008.03464.x 18315543

[kiad280-B30] Okawa K , Nakayama K, Kakizaki T, Yamashita T, Inaba T. Identification and characterization of Cor413im proteins as novel components of the chloroplast inner envelope. Plant Cell Environ. 2008:31(10):1470–1483. 10.1111/j.1365-3040.2008.01854.x 18643950

[kiad280-B31] Osakabe Y , Maruyama K, Seki M, Satou M, Shinozaki K, Yamaguchi-Shinozaki K. Leucine-rich repeat receptor-like kinase1 is a key membrane-bound regulator of abscisic acid early signaling in *Arabidopsis*. Plant Cell 2005:17(4):1105–1119. 10.1105/tpc.104.027474 15772289 PMC1087989

[kiad280-B32] Pandey S , Nelson DC, Assmann SM. Two novel GPCR-type G proteins are abscisic acid receptors in *Arabidopsis*. Cell 2009:136(1):136–148. 10.1016/j.cell.2008.12.026 19135895

[kiad280-B33] Shang Y , Yang D, Ha Y, Shin HY, Nam KH. Receptor-like protein kinases RPK1 and BAK1 sequentially form complexes with the cytoplasmic kinase OST1 to regulate ABA-induced stomatal closure. J Exp Bot. 2020:71(4):1491–1502. 10.1093/jxb/erz489 31665747

[kiad280-B34] Shi Y , Tian S, Hou L, Huang X, Zhang X, Guo H, Yang S. Ethylene signaling negatively regulates freezing tolerance by repressing expression of *CBF* and type-A *ARR* genes in *Arabidopsis*. Plant Cell 2012:24(6):2578–2595. 10.1105/tpc.112.098640 22706288 PMC3406918

[kiad280-B35] Shinozaki K , Yamaguchi-Shinozaki K, Seki M. Regulatory network of gene expression in the drought and cold stress responses. Curr Opin Plant Biol. 2003:6(5):410–417. 10.1016/S1369-5266(03)00092-X 12972040

[kiad280-B36] Shirakawa M , Morisaki Y, Gan ES, Sato A, Ito T. Identification of a devernalization inducer by chemical screening approaches in *Arabidopsis thaliana*. Front Plant Sci. 2021:12:634068. 10.3389/fpls.2021.634068 PMC7890032 33613612

[kiad280-B37] Stockinger EJ , Gilmour SJ, Thomashow MF. *Arabidopsis thaliana CBF1* encodes an AP2 domain-containing transcriptional activator that binds to the C-repeat/DRE, a cis-acting DNA regulatory element that stimulates transcription in response to low temperature and water deficit. Proc Natl Acad Sci U S A. 1997:94(3):1035–1040. 10.1073/pnas.94.3.1035 9023378 PMC19635

[kiad280-B38] Thomashow MF . So what’s new in the field of plant cold acclimation? Lots! Plant Physiol. 2001:125(1):89–93. 10.1104/pp.125.1.89 11154304 PMC1539333

[kiad280-B39] Tischer W , Strotmann H. Relationship between inhibitor binding by chloroplasts and inhibition of photosynthetic electron transport. Biochim Biophys Acta. 1977:460(1):113–125. 10.1016/0005-2728(77)90157-8 856261

[kiad280-B40] Tokumaru M , Adachi F, Toda M, Ito-Inaba Y, Yazu F, Hirosawa Y, Sakakibara Y, Suiko M, Kakizaki T, Inaba T. Ubiquitin-proteasome dependent regulation of the GOLDEN2-LIKE 1 transcription factor in response to plastid signals. Plant Physiol. 2017:173(1):524–535. 10.1104/pp.16.01546 27821720 PMC5210752

[kiad280-B41] Uehara TN , Mizutani Y, Kuwata K, Hirota T, Sato A, Mizoi J, Takao S, Matsuo H, Suzuki T, Ito S, et al Casein kinase 1 family regulates PRR5 and TOC1 in the *Arabidopsis* circadian clock. Proc Natl Acad Sci U S A. 2019:116(23):11528–11536. 10.1073/pnas.1903357116 31097584 PMC6561244

[kiad280-B42] Uemura M , Joseph RA, Steponkus PL. Cold acclimation of *Arabidopsis thaliana* (effect on plasma membrane lipid composition and freeze-induced lesions). Plant Physiol. 1995:109(1):15–30. 10.1104/pp.109.1.15 12228580 PMC157560

[kiad280-B43] Vogel JT , Zarka DG, Van Buskirk HA, Fowler SG, Thomashow MF. Roles of the CBF2 and ZAT12 transcription factors in configuring the low temperature transcriptome of *Arabidopsis*. Plant J. 2005:41(2):195–211. 10.1111/j.1365-313X.2004.02288.x 15634197

[kiad280-B44] Yamaguchi-Shinozaki K , Shinozaki K. Characterization of the expression of a desiccation-responsive rd29 gene of *Arabidopsis* thaliana and analysis of its promoter in transgenic plants. Mol Gen Genet. 1993:236–236(2–3):331–340. 10.1007/BF00277130 8437577

[kiad280-B45] Zhang H , Deng X, Miki D, Cutler S, La H, Hou YJ, Oh J, Zhu JK. Sulfamethazine suppresses epigenetic silencing in *Arabidopsis* by impairing folate synthesis. Plant Cell 2012:24(3):1230–1241. 10.1105/tpc.112.096149 22447685 PMC3336112

[kiad280-B46] Zhu JK . Cell signaling under salt, water and cold stresses. Curr Opin Plant Biol. 2001:4(5):401–406. 10.1016/S1369-5266(00)00192-8 11597497

