# Supplementary material for: Chemical screening approach using single leaves identifies compounds that affect cold signaling in Arabidopsis
Source: Plant Physiol. 2023 May 13;193(1):234–45. doi: 10.1093/plphys/kiad280 (PMC10469520; doi:10.1093/plphys/kiad280)
Supplement: kiad280_Supplementary_Data [file kiad280_supplementary_data.zip › Kitawaki Sup Figures and Tables combined.pdf]

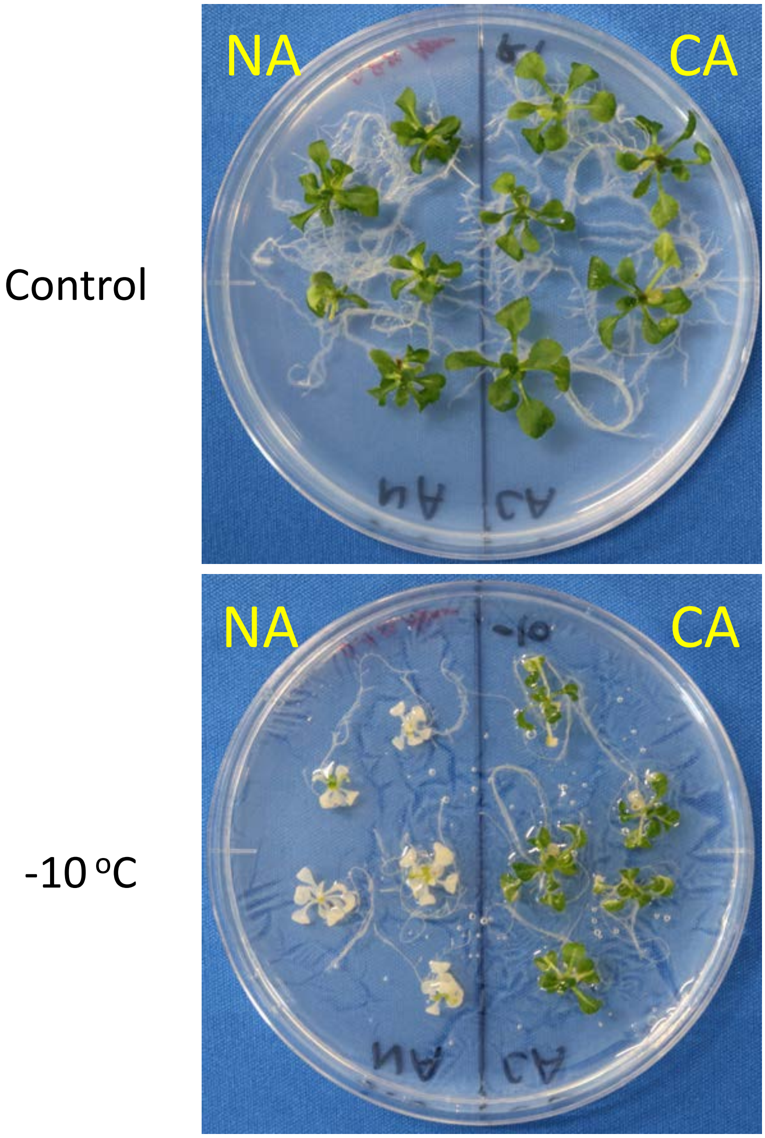

Supplemental Figure S1. Arabidopsis grown in submerged cultures can cold-acclimate and increase the freezing tolerance

Arabidopsis seedlings grown in submerged cultures for 11 days were cold-acclimated at 4°C (CA) or kept at 22°C (NA) for two days. Plants were then transferred to 1/2 MS plates containing 0.8% agar under sterile condition. Plates were incubated at -2°C for 2 h and then cooled down at a rate of 1°C per 30 min in the dark. After reaching to -10°C, plants were kept at -10°C for additional 1 h. Plants were then placed in the dark overnight at 4°C to thaw. The thawed plants were returned to growth chamber (22°C, continuous light) and allowed to grow for approximately 60 h. Controls represent plants that are returned to growth chamber without freezing stress after transfer from liquid MS to MS agar plates.

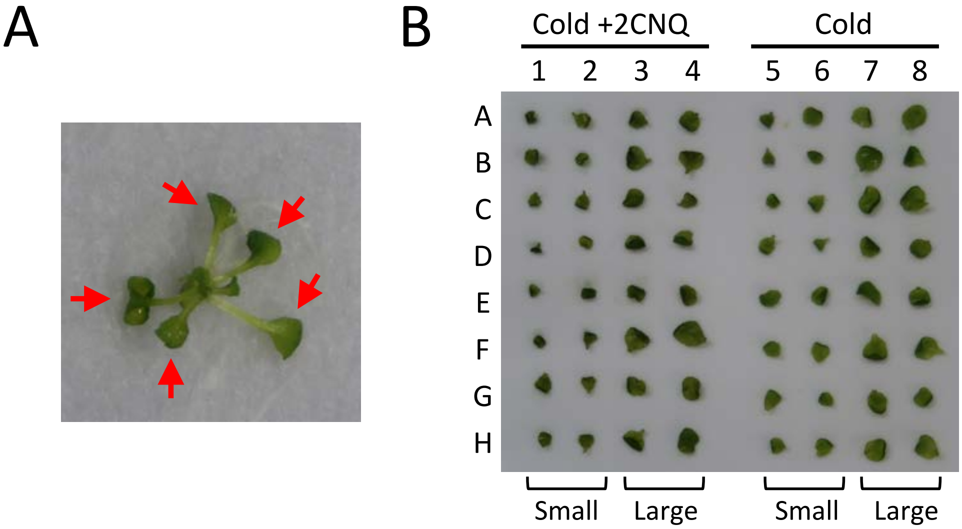

C

	Cold +2CNQ				Cold			
	1	2	3	4	5	6	7	8
A	54198	58990	2809	9883	4014540	2745920	4623700	1360220
B	27192	7625	5741	8789	2761870	980051	843706	734280
C	2053	8565	178090	25432	1386090	1196150	691849	2275490
D	5033	7887	9614	7313	2641390	1292760	2665520	525809
E	269563	8099	11168	6943	1631180	1034740	1472870	3260070
F	4991	4599	5960	9027	326748	5752030	1196270	678542
G	6616	171560	3804	9538	4372330	2351980	1367070	1046790
H	43586	1772	16763	93035	1323270	844144	1604140	4079940
	Small		Large		Small		Large	

Supplemental Figure S2. Effects of leaf size on *COR15A pro::LUC* activity
A, Photograph of *COR15Apro::LUC* plant grown in a submerged culture. Arrows indicate representative leaves used for high throughput screening.
B. Photograph of each leaf used for analysis. Excised leaves were divided into two classes depending on the size (large or small).
C. Direct measurement of luciferase activity derived from each leaf using a Varioskan Flash. Excised small and large leaves of *COR15Apro::LUC* plants grown in submerged cultures were treated with low temperature (Cold). Leaves were also treated with low temperature in the presence of 2-chloro-1,4-naphthoquinone (Cold + 2CNQ). 2CNQ, an inhibitor of *COR15Apro::LUC* induction (see Figure 4), was used to ensure that leaf size did not affect the effect of the inhibitor. The row and column positions are the same as those in panel B. Luciferase activity is shown as relative light unit (RLU).

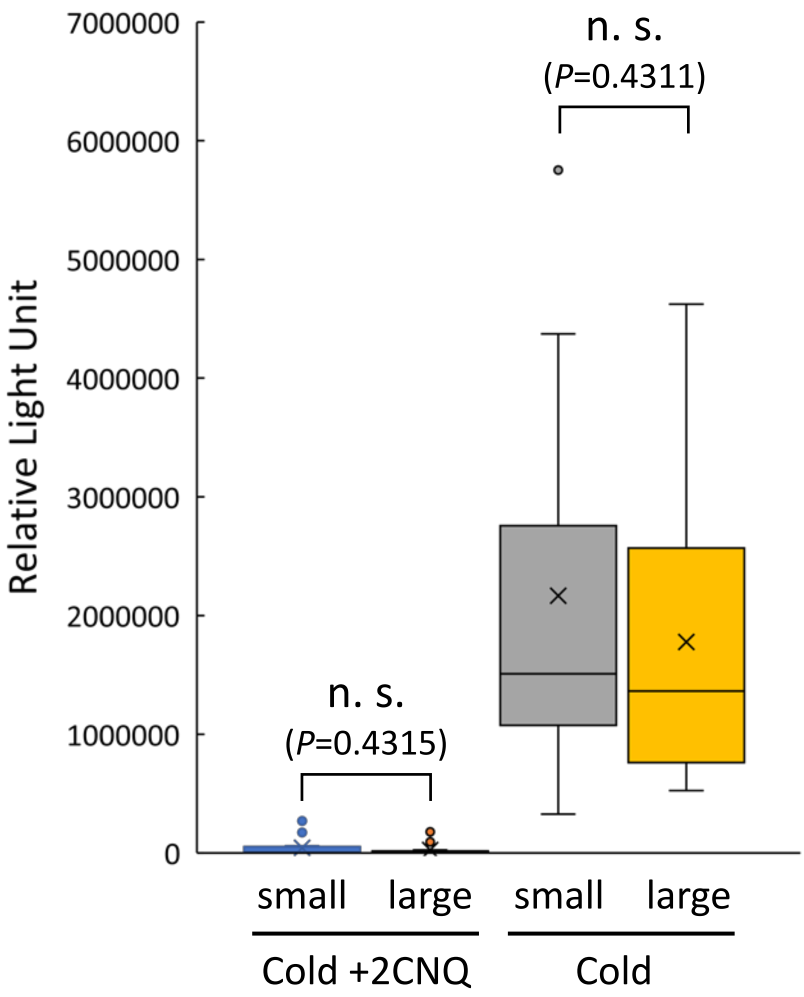

Supplemental Figure S3. Comparison of the effect of leaf size on the cold-induced luciferase activity in *COR15Apro::LUC* leaves using box-and-whisker plots

Graphs are box-and-whisker plots where the center horizontal indicates the median; upper and lower edges of the box are the upper and lower quartiles; and whiskers extend to the maximum and minimum values within 1.5 interquartile ranges. The x marks represent means, and dots indicate outliers. Graphs were drawn using raw data of relative light unit shown in Supplemental Figure S2C. Statistical difference between large and small leaves were analyzed by student's *t*-test. n.s., not significant.

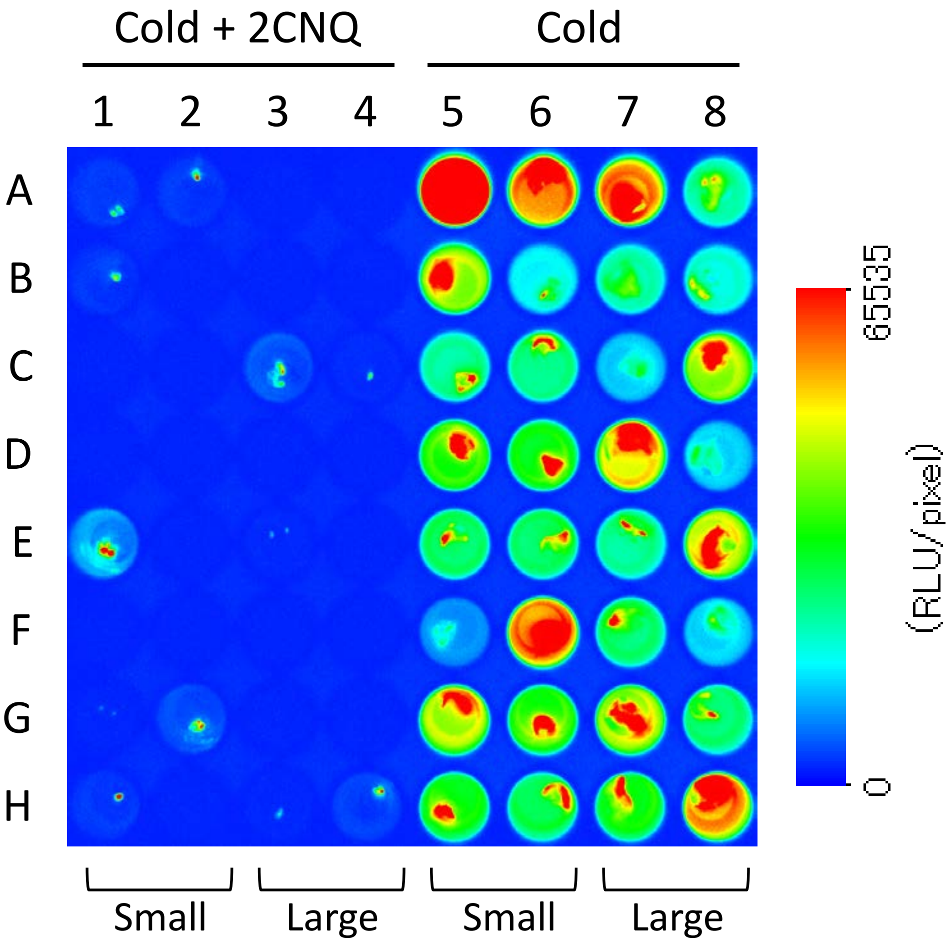

Supplemental Figure S4. Visualization of LUC activity in excised *COR15Apro::LUC* leaves using cooled CCD camera

LUC images were captured by cooled CCD imaging apparatus Luminograph II (ATTO Co. Ltd., Japan). The same set of leaves shown in Supplemental Figure S2B were used, and the row and column positions are the same as those in Supplemental Figure S2B. 2CNQ, an inhibitor of *COR15Apro::LUC* induction, was used to ensure that leaf size did not affect the effect of the inhibitor.

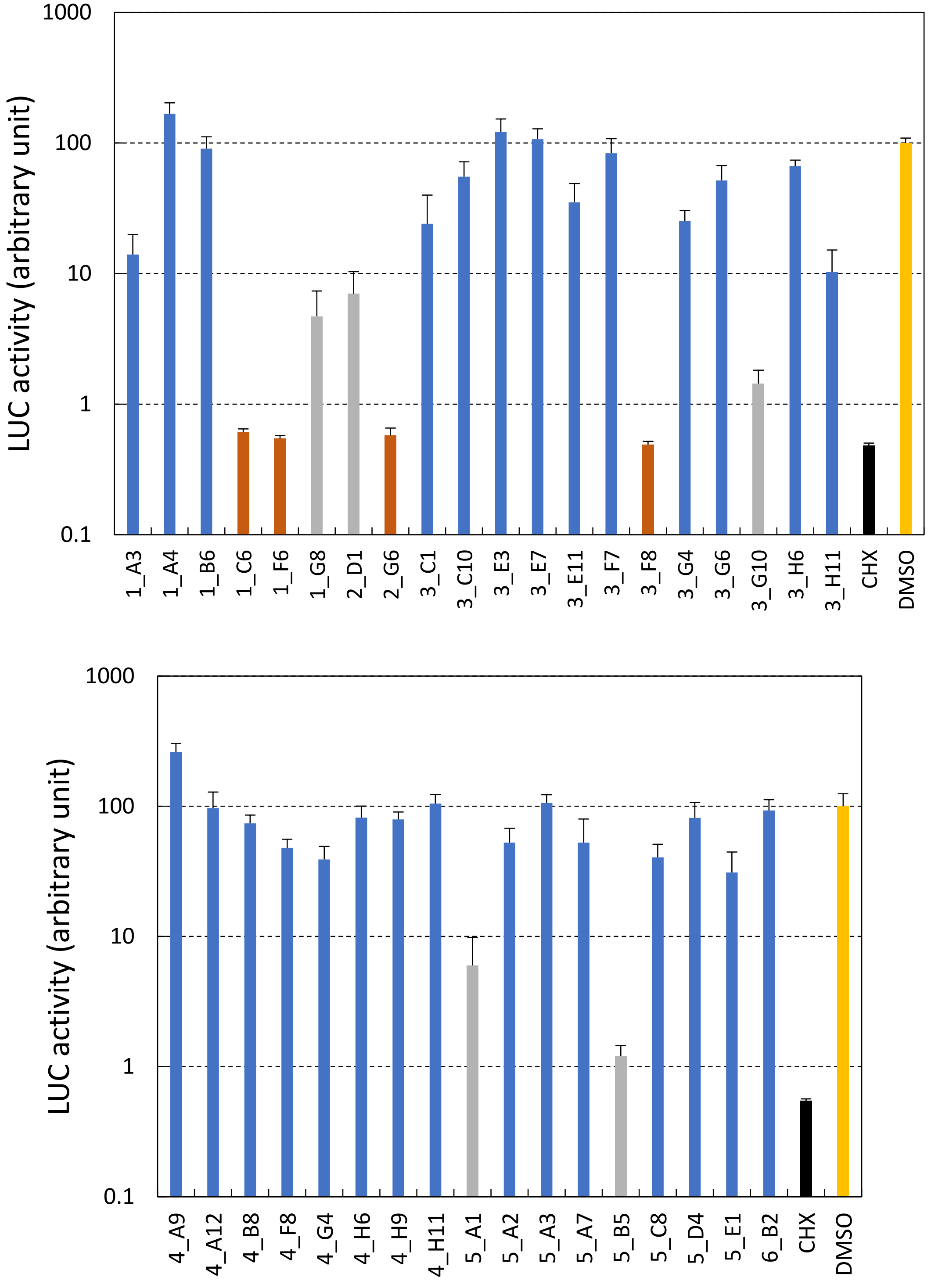

Supplemental Figure S5. Second screening for the identification of natural compounds that inhibit luciferase activity of *COR15Apro::LUC*

Natural compounds that was identified by the first screening were further investigated. The number on the X axis is the identification number of each natural compound in chemical library plates. Luciferase activity of DMSO-treated plants was set to 100. Error bars represent standard errors of the mean (*n*=4).

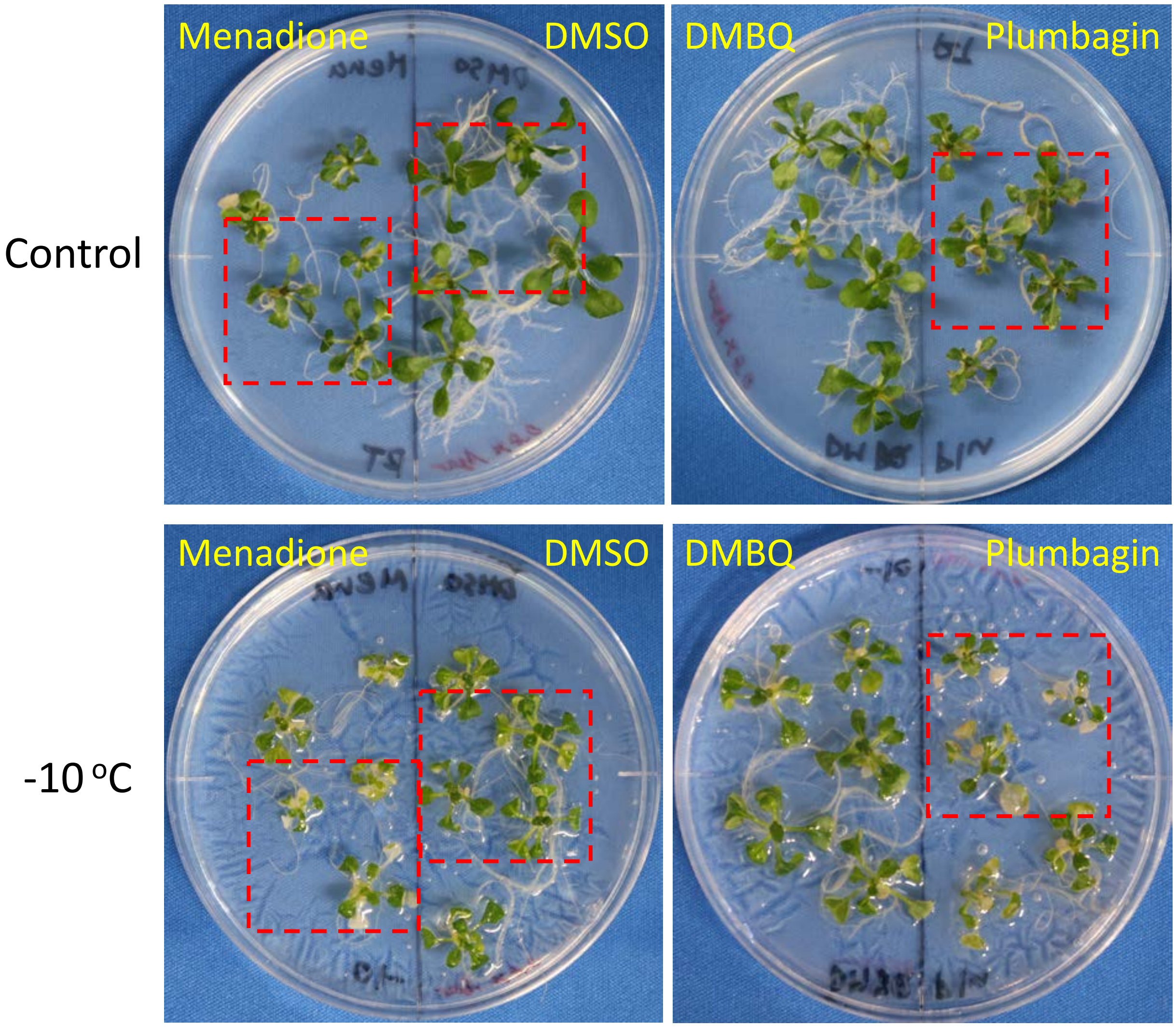

Supplemental Figure S6. Freezing tolerance of Arabidopsis cold-acclimated in the presence of DMSO, menadione, plumbagin or DMBQ.
Arabidopsis seedlings grown in submerged cultures were cold-acclimated for two days in the presence of DMSO, menadione, plumbagin or DMBQ. Plants were then transferred to 1/2 MS plates containing 0.8% agar under sterile condition. Plates were incubated at -2°C for 2 h and then cooled down at a rate of 1°C per 30 min in the dark. After reaching to -10°C, plants were kept at -10°C for additional 1 h. Plants were then placed in the dark overnight at 4°C to thaw. The thawed plants were returned to growth chamber (22°C, continuous light) and allowed to grow for approximately 60 h. Controls represent plants that are returned to growth chamber without freezing stress. Dashed boxes indicate plants that were shown in Figure 3C.

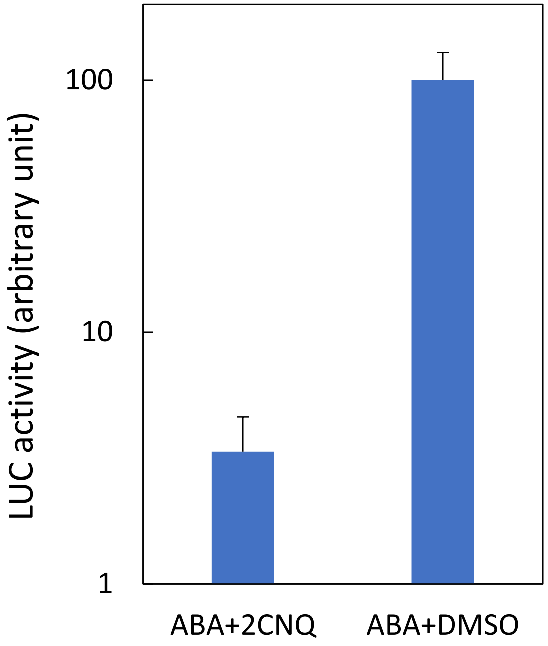

Supplemental Figure S7. Induction of *COR15Apro::LUC* activity by ABA is strongly inhibited by simultaneous treatment with 2-chloro-1,4-naphthoquinone.
Excised leaves of *COR15Apro::LUC* plants grown in submerged cultures were treated with ABA for 4 h in the presence of 2-chloro-1,4-naphthoquinone (2CNQ) or DMSO. Luciferase activity was measured using a Varioskan Flash microplate reader. Error bars represent standard errors of the mean (*n*=16).

Supplemental Table S2: List of potent inhibitors that affect cold induction of *COR15Apro:LUC*

Plate Location	Common Name	Possible Function
1-C6	Cycloheximide	Inhibitor of eukaryotic protein synthesis.
1-F6	Gliotoxin	Inhibitor of 20S proteasome.
2-G6	Menadione	Inducer of apoptosis. Vitamin K3.
3-F8	Plumbagin	Inducer of cell cycle arrest, apoptosis, and autophagy.

Supplemental Table S3. Primers used for construction of *COR15Apro::LUC*

Amplified fragment	Primers	
COR15A promoter	Forward	5’-TGATTACGCCAAGCTTGTCCGTTGAATTTATTTTAGACT-3’
	Reverse	5’-TTTGGCGTCTTCCATGAGAGAGATCTTTAAGATGTGAGAA-3’
Luciferase (LUC)	Forward	5’-ATGGAAGACGCCAAAAACATAAAG-3’
	Reverse	5’-GATCGAATTCTCTAGATTACACGGCGATCTTTCCGCCCTTC-3’

Supplemental Table S4. Gene-specific primers used in RT-qPCR

Gene Name	AGI code	Forward Primer	Reverse Primer
ACTIN2	At3g18780	5'-GCACCCTGTTCTTCTTACCG-3'	5'-AACCCTCGTAGATTGGCACA-3'
COR15A	At2g42540	5'-GTATGTGGAGGAGAAAGGAA-3'	5'-AACTGATTAGGTAAGACCCTAC-3'
COR413IM1	At1g29395	5'-GCAAGAAGGGGCAATCATAGCAA-3'	5'-TCTCATATGCTTTCTCCAAGCTC-3'
KIN2	At5g15970	5'-GAGTATATCGGATGCGGCAGTG-3'	5'-AGTTGACTCGGATCGCTACTTG-3'
RD29A	At5g52310	5'-CGGTGCAGAAGGAGCTTTAAG-3'	5'-CTGGTATGGAGGAACTTCTTAATCC-3'
CHS	At5g13930	5'-CGTGTTGAGCGAGTATGGAAAC-3'	5'-TGTTTAGAGAGGAACGCTGTGC-3'
PDH-E1α	At1g01090	5'-TGCAAAGGAAGCAGAGCTAAAG-3'	5'-CCTCACATCTGTACCGTCCATC-3'
CBF1	At4g25490	5'-GGAGACAATGTTTGGGATGC-3'	5'-CGACTATCGAATATTAGTAACTCCAAAGCGACACG-3'
CBF2	At4g25470	5'-GACGTGTCCTTATGGAGCTATTAAAA-3'	5'-TTACCATTTACATTCGTTTCTCACAAC-3'
CBF3	At4g25480	5'-TTCCGTCCGTACAGTGGAAT-3'	5'-AACTCCATAACGATACGTCGTC-3'
ZAT12	At5g59820	5'-TCGTCGCATCCTTGTCCCAT-3'	5'-AACGTAGTCACCGTGGGCTC-3'
MYB15	At3g23250	5'-AGCCCTCCCTAAGCAAGCTG-3'	5'-TGCTGCAATCGCTGACCATC-3'
EIN3	At3g20770	5'-TGGGAAGCCTGTGACTGGTG-3'	5'-GGAGTCGGTCCAATCGGGTT-3'
